# RNAi screening identifies *Trypanosoma brucei* stress response protein kinases required for survival in the mouse

**DOI:** 10.1038/s41598-017-06501-8

**Published:** 2017-07-21

**Authors:** Fernando Fernandez-Cortes, Tiago D. Serafim, Jonathan M. Wilkes, Nathaniel G. Jones, Ryan Ritchie, Richard McCulloch, Jeremy C. Mottram

**Affiliations:** 1 Wellcome Centre for Molecular Parasitology and Institute of Infection, Immunity and Inflammation, College of Medical, Veterinary and Life Sciences, University of Glasgow, Glasgow, UK; 2 Centre for Immunology and Infection, Department of Biology, University of York, Wentworth Way, Heslington, York UK; 3 Present Address: Vector Molecular Biology Section, Laboratory of Malaria and Vector Research, National Institute of Allergy and Infectious Diseases, National Institutes of Health, Rockville, MD USA

## Abstract

Protein kinases (PKs) are a class of druggable targets in *Trypanosoma brucei*, the causative agent of Human African Trypanosomiasis (sleeping sickness), yet little is known about which PKs are essential for survival in mammals. A recent kinome-wide RNAi screen with 176 individual bloodstream form *Trypanosoma brucei* lines identified PKs required for proliferation in culture. In order to assess which PKs are also potential virulence factors essential *in vivo*, lines were pooled, inoculated into mice, and screened for loss of fitness after 48 h RNAi. The presence of trypanosomes in the bloodstream was assessed using RNAi target sequencing (RITseq) and compared to growth in culture. We identified 49 PKs with a significant loss of fitness *in vivo* in two independent experiments, and a strong correlation between *in vitro* and *in vivo* loss of fitness for the majority. Nine PKs had a more pronounced growth defect *in vivo*, than *in vitro*. Amongst these PKs were several with putative functions related to stress responses mediated through the PI3K/TOR or MAPK signaling cascades, which act to protect the parasite from complement-mediated and osmotic lysis. Identification of these virulence-associated PKs provides new insights into *T. brucei*-host interaction and reveals novel potential protein kinase drug targets.

## Introduction

*Trypanosoma brucei* is an obligate parasite in the class kinetoplastida, a group of flagellated protozoa characterized by a dense complex of circular mitochondrial DNA called the kinetoplast. *T. brucei spp* infect humans and animals in sub-Saharan Africa, causing diseases that remain major threats to health and economies in the region. *T. brucei* exhibits a complex life cycle that requires adaptation to many different environments, including different compartments within the blood-feeding insect vector responsible for transmission between different mammalian hosts: the tsetse fly. Once in the mammal, *T. brucei* resides extracellularly in the bloodstream, tissue fluids, central nervous system and adipose tissue^1^. Bloodstream *T. brucei* undergo antigenic variation and express a single Variant Surface Glycoprotein (VSG) per cell, which can be switched upon expansion of the population to create diversity^2^. The host builds an adaptive immune response against at least the most abundant variants, leading to their clearance and enabling outgrowth of cells that have switched to an antigenically distinct VSG. Iteration of this process leads to the characteristic waves of parasitemia^3^.

Protein kinases (PKs) are key signalling proteins in eukaryotes, playing critical roles as central regulators in many biological functions, as well as being validated drug targets. The *T. brucei* protein kinome represents 2% of the parasite’s protein-coding capacity and comprises 157 conserved eukaryotic PKs (ePKs), 12 non-catalytic pseudokinases and 20 atypical PKs (aPKs)^4–6^. Substantial differences exist between the *T. brucei* and the human protein kinomes, as the parasites lack receptor-linked tyrosine kinases and tyrosine-like kinases. Despite this, tyrosine phosphorylation has been reported, possibly due to dual-specificity kinases^4, 5^. *T. brucei* also has a relatively reduced representation of AGC and CAMK families, while CMGCs, STEs and NEKs are comparatively expanded. In addition, several highly divergent PKs are likely to play parasite-specific functions that may present targets for selective inhibition by small molecules^4, 5^.

PKs are a promising source of druggable targets, with more than 100 inhibitors already in clinical trials and successful drugs in the market, such as the prototypical compound Imatinib® for chronic myeloid leukemia^7^. Several high-throughput screening campaigns with compound libraries have been published linking *in vitro* trypanocidal activity with PK inhibition^8–10^, though the specific PK target in each case is unknown. Over 40 *T. brucei* PKs have been shown to be essential for normal cell proliferation *in vitro* ^6, 11^. Many biological roles for PKs have been revealed in *T. brucei*, but they have been observed mainly in the artificial conditions of *in vitro* culture. In this paper we use a kinome-focused RNAi library in a 72 h mouse infection model to address a key question of both biological and pharmaceutical relevance: which PKs are required for survival of the parasite in the environment of the mammalian bloodstream?

## Results

### Kinome-wide *in vitro* and *in vivo* RNAi screens

**Figure 1:**
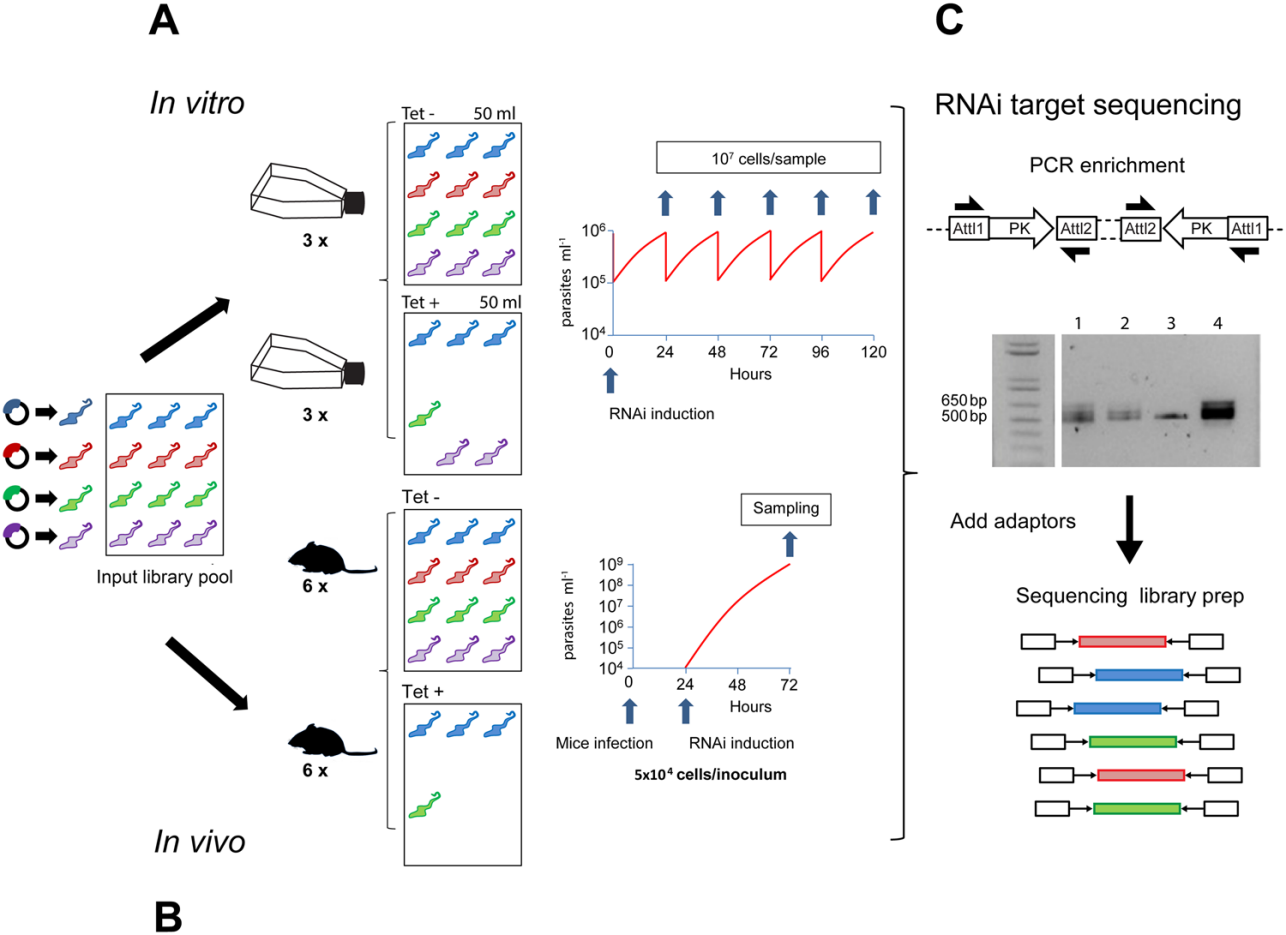
*In vitro* and *in vivo* phenotyping of a *T. brucei* kinome RNAi library. Schematic representation of the experimental workflow. (**A**) A pre-inoculation pooled kinome RNAi library was diluted to contain 1 × 10^5^ cell ml^−1^ in 100 ml and grown in culture for 24 h in triplicate. Each culture was then split into two flasks, one in which RNAi was induced with tetracycline (Tet+) and the other remained uninduced (Tet−). 1 × 10^7^ cells were sampled every 24 h over 5 days for DNA purification and cultures diluted daily to contain 1 × 10^5^ cells ml^−1^. (**B**) 5 × 10^4^ *T. brucei* bloodstream form parasites of the pooled kinome RNAi library were injected intraperitoneally into 12 CD1 mice and 24 h post inoculation, RNAi was induced with doxycycline in 6 animals (Tet+ 1–6) and 6 were left uninduced (Tet− 1–6). 48 h post RNAi induction, parasites were purified from blood and genomic DNA prepared. (**C**) PCR enrichment of the RNAi target was carried out. The cropped gel example shows RNAi target distribution in 4 different samples: Tet− (1), Tet+ (2), a single cell line control (3), and the preinoculation pool (4). The experiment was performed twice and PCR-enriched samples from each biological replicate were sequenced. A full-length gel is presented in Figure S5.

We had previously generated a collection of individual *T. brucei* RNAi cell lines to identify PKs essential for proliferation of bloodstream form parasites in culture, cell cycle regulators and negative regulators of BSF to PCF differentiation^6^. In order to increase the capacity for screening the kinome RNAi library, we made a pool of the 177 available cell lines, which targeted 183 of the PKs (6 were double knockdowns)^6^. This pool allowed parallel phenotyping of the population in a single culture (*in vitro*) and in a single mouse (*in vivo*) through RNAi target sequencing (Fig. 1). Cytocidal or cytostatic phenotypes were identified by a quantitative reduction in the reads mapping to a particular RNAi target in the induced samples (tet+) relative to their expanded representation in the uninduced (tet−) controls.

**Figure 2:**
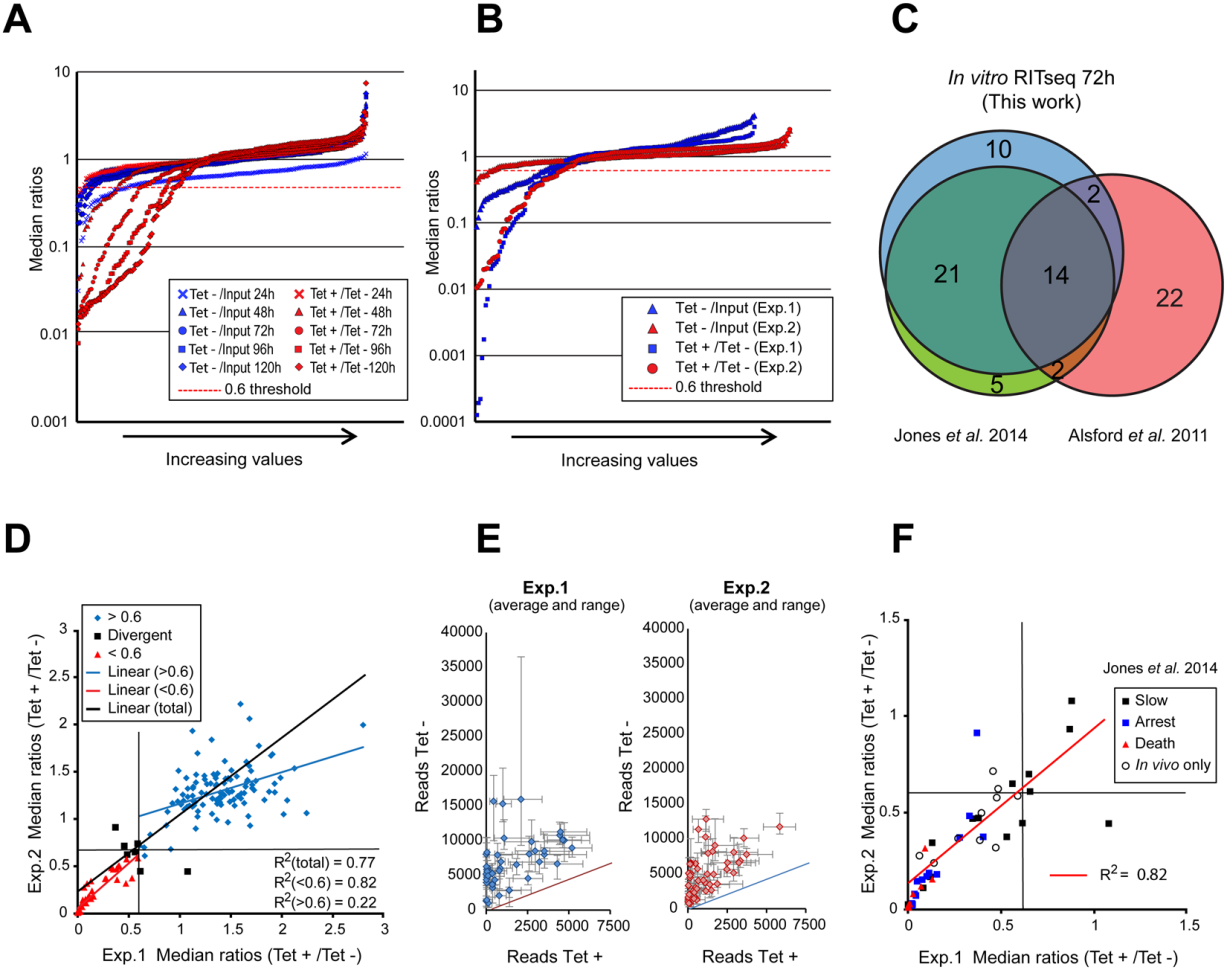
Intrinsic reproducibility and correlation between *in vivo* and *in vitro* RITseq. (**A**) Bootstrap median ratios of uninduced/input (Tet−/input) and induced/uninduced (Tet+/Tet−) in triplicate for each time point of the *in vitro* RNAi screen, arranged in increasing order. Reads were normalized as reads per million (RPM). (**B**) Bootstrap median ratios of uninduced/input (Tet−/input) and induced/uninduced (Tet+/Tet−) of each *in vivo* experiment arranged in increasing order. (**C**) Venn diagram showing the overlap between the number of loss-of-fitness PK depletions identified with the kinome-wide *in vitro* RITseq screen after 72 h of RNAi induction and the two *in vitro* studies covering the PKs of *T. brucei* available in the literature^6, 12^ at the same time point. (**D**) Bootstrap medians of ratios calculated between induced and uninduced *in vivo* samples plotted per RNAi target assessing regression of both biological replicates. Highlighted in red are those with a loss of fitness in the two experiments, in black those with a loss of fitness in one only, and blue those without a loss of fitness. (**E)** Breakdown of results for loss of fitness RNAi lines within each of the individual *in vivo* biological replicates. Depicted average and range of normalized counts for the 6 mice analyzed per condition (Tet+ or Tet−) and per RNAi target. (**F**) As panel D but focusing on the RNAi cell lines giving a loss of fitness (ratio < 0.6) in at least one of the 2 biological replicates. Includes depicted correlation with the *in vitro* screen previously published by Jones *et al*.^6^ with the same library (slow growth, cell cycle arrest or death), highlighting PKs whose depletion produced loss of fitness only *in vivo*.

For both the *in vitro* and the *in vivo* experiments, the reads were filtered to include only those that contained a 9-nucleotide tag encoded in the PCR primers and were then mapped against the sequences of *T. brucei* PK ORFs. Ratios were determined from the reads that mapped to each PK gene in the induced sample relative to the uninduced sample (and in the uninduced relative to the pre-inoculation pool) with a bootstrap analysis including 1000 repeats, which enabled calculation of the median and estimation of 95% confidence intervals for each gene (Data sets S2–S3). Ratios under 0.6 were considered loss of fitness phenotypes based on the scores for the PK with an upper limit for the 95% confidence interval under 0.75 in the biological replicates performed *in vivo*. When the 0.6 (induced/uninduced) threshold was applied to the *in vitro* data set it was determined to be discriminatory, showing a progressive increase of the number of targets identified with time (Fig. 2A), and a comparable profile in the *in vivo* dataset (Fig. 2B).

For the *in vitro* RITseq screen, the results of read mapping (Data sets S1 and S2) show an increase with time of both the number of PKs that displayed loss of fitness (as defined by induced/uninduced ratios < 0.6, Fig. 2A), and the extent of loss of individual PKs from the population (Data set S1). Comparing the *in vitro* screen with available data in the literature, 38 out of the 47 lines found at the 72 h time point had also been identified in the 72 h *in vitro* Alamar blue screen performed by Jones *et al*.^6^ with the same cell lines (Fig. 2C). Overlap with the genome-wide RITseq.^12^ was less pronounced, with only 18 of the PKs identified in this study being detected, notably those with the most deleterious growth phenotypes.

The *in vivo* experiment was repeated to provide two biological replicates, with the PCR products barcoded, multi-plexed and sequenced using two different platforms: Ion Torrent^TM^ and Illumina^®^. A total of 157 RNA cell lines were detected in the first *in vivo* experiment, meaning 24 cell lines were missing, possibly due to loss of individual cell lines during amplification of the pre-inoculation pool. The missing PK RNAi cells were remade and added to the second experiment, enabling detection of 177 PKs. Four PKs were excluded from the analysis due to low abundance in the initial inoculum, which made assignment of reads inaccurate (Data set S3). Analyzing the *in vivo* data based on the 0.6 induced/uninduced threshold, 53 PK genes were identified that showed a relative depletion of reads in the induced samples, indicating a loss of fitness 48 h after RNAi induction in one or both of the experiments. Forty genes showed such loss of fitness in both of the biological replicates (Data set S1), with six seen only in Experiment 1 and eight only in Experiment 2 (six of which were absent in the first experiment).

Linear regression across the two *in vivo* experiments showed good reproducibility for the complete dataset (R^2^ = 0.77; p < 0.0001, Fig. 2D), and substantially better if only the 49 RNAi targets with an average induced/uninduced ratio < 0.6 between the 2 biological replicates were compared (R^2^ = 0.82; p < 0.0001, Fig. 2D). The regression value for cell lines with fitness ratios over the 0.6 threshold decreased to R^2^ = 0.22. The intrinsic reproducibility across mice for both uninduced and induced groups per gene identifier was also excellent within the two biological replicates (see means and ranges displayed in Fig. 2E). Thirty-five out of 42 RNAi lines displaying loss fitness in the Jones screen also had a loss of fitness *in vivo*, with the most pronounced ‘death’ or ‘cell cycle arrest’ phenotypes *in vitro* also having the most significant loss of fitness *in vivo* (Figs 2F and S1A). Among the seven RNAi lines displaying significant loss of fitness *in vitro* but not *in vivo*, six were classified as “slow growth” and they only had significant growth retardation after 72 h of RNAi induction, which would not have been apparent in the 48 h timescale of the *in vivo* screen. In order to validate the observed correlation between the published *in vitro* loss-of-fitness phenotypes and those observed *in vivo*, growth analysis of 4 cell lines showing no growth defect or slow growth, growth arrest or death (as defined in ref. 6) were performed (Fig. S1B). A good correlation was observed between the *in vitro* growth and the median ratios of induced/uninduced for the *in vivo* RITseq. Comparing the PKs that showed loss of fitness *in vitro* and *in vivo* revealed notable overlap, though it was clear that there was a delay in the manifestation of loss of fitness *in vitro* when compared with the *in vivo* infection (Fig. S1C).

### Classification of protein kinases required for optimal growth ***in vivo*** after 48 h of induction

**Figure 3:**
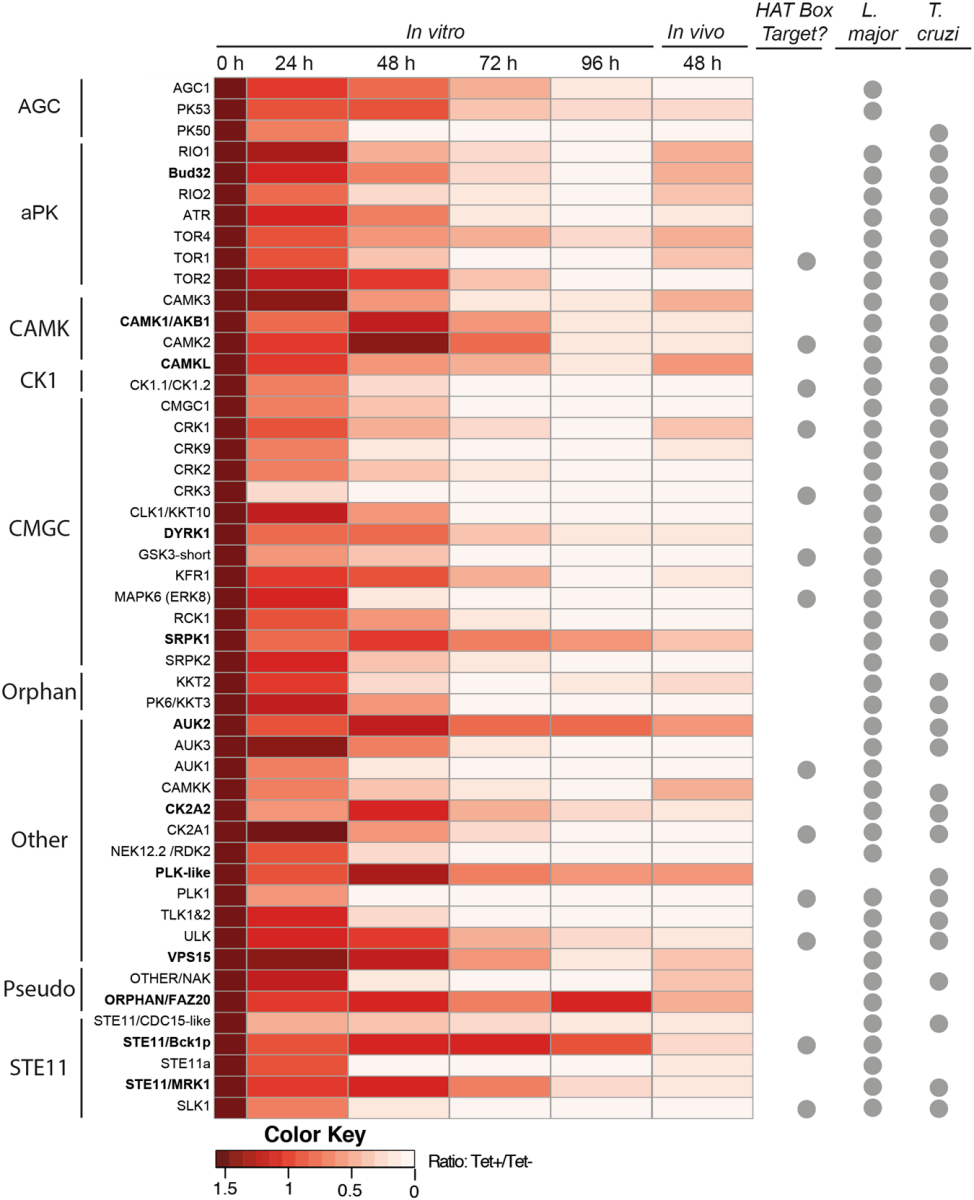
Heatmap depicting 49 cell lines showing loss of fitness during *in vivo* RNAi and their corresponding *in vitro* RNAi phenotypes. An individual PK from the library is represented in each row and they are grouped by family; those appearing in bold font were not identified as showing loss of fitness by *in vitro* Alamar blue screening^6^. The 0 h column is artificially adjusted to a solid colour representing a baseline value, the remaining heatmap columns represent the actual mean RIT-seq value determined at each time point up to 96 h *in vitro* and the 48 h *in vivo* time point. Lighter shades represent the reduction in the RIT-seq value and thus the loss of a cell line from the library. *HAT Box Target?* refers to a protein kinase that is a potential target for compounds in the GSK HAT Box by phylogenetic orthology to a human PK targeted by a compound in the box^8^. The *L. major* and *T. cruzi* columns use a circle to denote if an orthologue of the *T. brucei* gene encoding the protein kinase is present in the respective organism (see Data set S1).

The 49 PKs whose depletion caused an *in vivo* loss of fitness are distributed across all the major PK families (Fig. 3 and Data set S1). There are 40 ePKs, 7 aPKs and 2 putative pseudo-kinases. Two ePKs and one of the pseudo-kinases are considered “orphan” (without clear identity with known orthologues in *Saccharomyces cerevisiae*, *Caenorhabditis elegans, Drosophila melanogaster* or *Homo sapiens)* and are likely to be kinetoplastid-specific. All of the 49 PKs have orthologues in either *Leishmania major* or *Trypanosoma cruzi* or both, though with variable levels of sequence identity between the kinetoplastid parasites (Figs 3 and S2, Data set S1). Thirty-five of the PKs that were identified in the Alamar blue kinome-wide RNAi screen have been discussed previously^6^, and so details will not be provided here. Three of these genes were identified in the whole-genome RITseq.^12^: PLK1 and KKT2, which have been shown to be essential in other studies^13, 14^, and AUK2, which only showed a loss of fitness *in vitro* after 120 h of induction (Data set S1). Another PK with proven kinase activity, Tb927.10.14770 (CAMK, AKB1) has been reported to downregulate cytokinesis and cell division if overexpressed or downregulated^15^.

### Protein kinases with more pronounced loss of fitness ***in vivo*** than ***in vitro***

There were 12 PKs identified with a significant loss of fitness *in vivo* that were not reported in the *in vitro* alamar blue screen performed previously^6^, suggesting an important role for survival in the mammalian bloodstream. Two of these RNAi PK lines, targeting AKB1 and MRK1, were found to be missing from the original library. AKB1 depletion has been shown to result in a loss of fitness *in vitro* ^15^, however, both AKB1 and MRK1 depletion show a defect only after 96 h of RNAi induction in culture (Fig. 3). In contrast, AUK2 has been reported to have a loss of fitness in the whole genome RITseq screen^12^. While required *in vivo* from 48 h onwards, AUK2 did not show a phenotype until 120 h of induction *in vitro*. CAMKL showed loss of fitness from 48 h onwards and four other PKs from 72 h onwards: Bud32, VPS15, DYRK1 and CK2A2. SRPK1 showed loss of fitness from 96 h, whilst PLK-like and FAZ20 after 120 h. The only PK that did not show an *in vitro* phenotype at all was STE11/Bck1p.

In order to add an extra layer of validation, independent growth curves were performed in culture for individual RNAi cell lines (Fig. S3). DYRK (Tb927.7.3880) had a clear growth defect after 48 h of RNAi *in vitro* (Fig. S3A), and so this gene was not considered further. For the other PK genes, where no growth defect was detected after 48 h of RNAi induction *in vitro*, parasitemia was assessed for 72–96 h in mice, with or without induction of RNAi, after inoculation of each individual selected cell line (Fig. S3B). Tb927.11.9290 (FAZ20), Tb927.2.1820 (CAMKL), Tb927.11.850 (Bud32) and Tb927.11.9190 (VPS15) displayed some evidence for growth retardation after RNAi *in vitro* (Fig. S3B) but, in each case, the extent of growth impairment or death was more pronounced *in vivo*. For each of the remaining six cell lines there was no evidence of RNAi-induced growth retardation *in vitro*. Two of the lines, targeting Tb927.3.3920 (AUK2) and Tb927.10.10350 (STE11/Bck1p), experienced growth retardation *in vivo* from 48 h, indicating a loss of fitness. For three other genes, Tb927.2.2430 (Other/CK2A2), Tb927.7.960 (CMGC/SRPK1) and Tb927.10.14300 (STE11/MRK1), the loss of fitness after *in vivo* RNAi was even more pronounced, since a decrease in parasitemia or clearance was seen before 96 h (Fig. S3B).

Taken as a whole, these growth curves validate the kinome-wide RITseq both *in vivo* and *in vitro* for nine of twelve PKs predicted to play a more important role in the mammalian infection than in *in vitro* culture.

### Increased susceptibility of RNAi mutants to serum or osmotic stress

**Figure 4:**
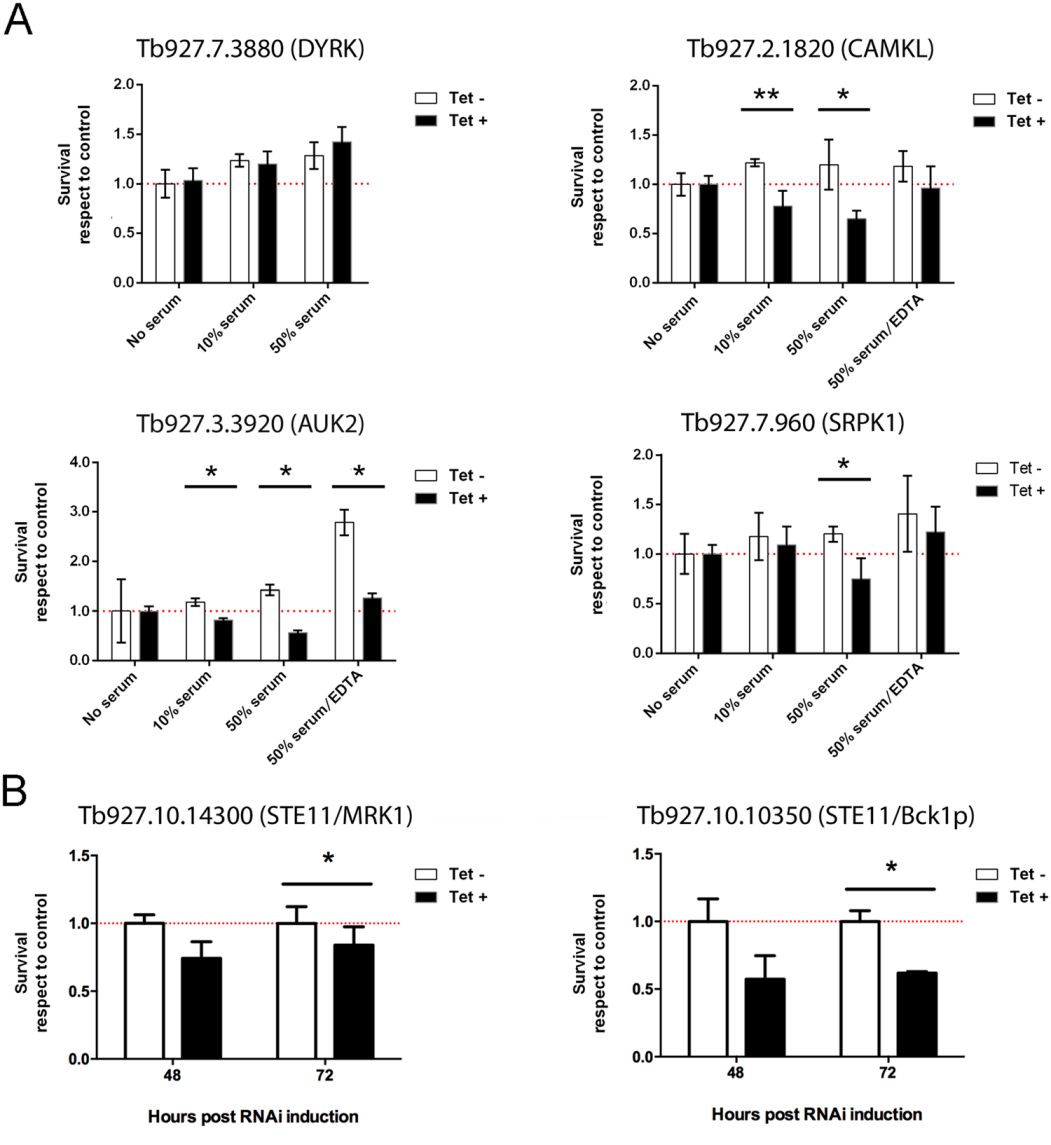
Survival when exposed to serum or osmotic shock. **(A**) Parasites at 10^6^ cells ml^−1^ were exposed for 3 h to rat serum (10% and 50% concentration). Relative survival was measured for induced (tet+, 72 h RNAi) and uninduced (tet−) clones compared to no-serum controls. Where deficient survival was detected in tet+, after addition of tet, cells were exposed to 50% serum complement-inactivated with 20 mM EDTA. (**B**) Cells were subjected to an osmotic shock protocol and survival of the induced cells (tet+, 48 h and 72 h RNAi) compared to uninduced (tet−) was plotted. *p < 0.05, using paired T-test.

One of the most obvious and distinctive elements encountered by parasites during infection in mice when compared with *in vitro* culture conditions is the exposure to mammalian serum factors, with the VSG coat providing protection against complement-mediated lysis^3^. To test if loss of potential PK virulence factors might impede survival in the presence of serum, we exposed PK RNAi lines shown to be defective in DNA repair (Stortz *et al.* ^16^, manuscript in press) to fresh rat serum for 3 h (10% and 50% concentration) and analyzed relative survival of RNAi induced and uninduced cells when compared to controls that were not exposed to rat serum (Fig. 4A). In addition, we tested Tb927.7.3880 (DYRK) that was found to produce a growth defect both *in vivo* and *in vitro*. Uninduced cells showed an increase in survival as serum concentration increased. RNAi against Tb927.7.3880 (DYRK), which caused impaired cell growth *in vitro*, caused no serum sensitivity. In contrast, RNAi against Tb927.2.1820 (CAMKL), Tb927.3.3920 (AUK2) and Tb927.7.960 (CMGC/SRPK1) resulted in significant, concentration-dependent sensitivity to serum, manifest in survival rates 50% lower than the equivalent uninduced samples. To understand whether such sensitivity was due to complement-mediated lysis, these three cell lines -RNAi induced and uninduced- were further exposed to 50% serum complement-inactivated with 20 mM EDTA^17^. For Tb927.2.1820 (CAMKL) and Tb927.7.960 (CMGC/SRPK1), the EDTA treatment ablated the serum sensitivity, strongly suggesting that the RNAi phenotype was due to increased complement-mediated killing. AUK2, however, was more difficult to interpret as Tet− cells showed increased survival when EDTA was added.

Two MAP3K PKs (Tb927.10.14300 and Tb927.10.10350) have a significantly more pronounced loss of fitness phenotype *in vivo* than *in vitro* (Fig. S3); these have sequence identity to yeast PKs that regulate osmotic stress (Data set S4). To test whether they might be involved in resistance to osmotic changes encountered during circulation in the mammalian host, a ‘Swell dialysis’ assay was performed^18^. Controlled buffering conditions were used to produce mildly turgid cells and assess how RNAi-induced cells survived gentle osmotic shock compared with uninduced controls (Fig. 4B). RNAi against each predicted STE11 PK resulted in a statistically significant reduction in cell survival in these conditions. RNAi of Tb927.10.14300 resulted in a 20% reduction in cell viability after osmotic shock at both 48 h and 72 h after RNAi induction. Ablation of Tb927.10.10350 resulted in a much more severe phenotype, with a 50% reduction in cell viability after osmotic shock at 48 h and 72 h after RNAi induction.

## Discussion

In this study we aimed to define the cohort of protein kinases that are essential for *T. brucei* to survive in the mammalian bloodstream. We modified whole-genome parallel phenotyping based on RITseq technology^12^ and applied it to a defined collection of individually designed and validated RNAi cell lines. This method was used to analyze *in vitro* and *in vivo* experiments to permit the identification of 49 essential PKs, including 9 PKs that appear more important for survival in the environment of the mammalian circulatory system than in culture. Bioinformatic assessment of each gene suggested these PKs are involved in parasite stress-response pathways. Validation of individual cell lines identified PKs that play roles in protecting the parasite from mammalian serum factors and osmotic stresses. Our findings expand the repertoire of essential and potentially druggable PKs in this pathogen and begin to explore the mechanisms by which the parasite survives the multiple stresses of life inside its host.

The adaptation of genome-wide RITseq technology to smaller, defined libraries was advantageous for *in vivo* experiments for several reasons. The coefficient of determination between the induced/uninduced read mapping ratios obtained between the two experiments was very good, especially when focused on those with a loss of fitness RNAi phenotype (Fig. 2F). Intrinsic reproducibility across different mice within the same experiment was also excellent (Fig. 2E). Inclusion of a single clone per RNAi target may allow an advantage in terms of phenotypic homogeneity compared with strategies where a pool of RNAi vectors are transfected into parasites to generate an unknown number of clones per target with different levels of depletion after induction. We show that focusing RITseq to smaller libraries^6^ enhances resolution of the loss of fitness readout enabling a ~1000-fold increase in sequence read depth in comparison to the whole genome screen (Fig. S4). This increase in resolution meant that we were able to detect 56 PKs that result in a loss of fitness upon depletion with the kinome-library *in vivo* or *in vitro*, while only 19 of these PKs with the most severe growth defects (Data set S1) were identified in a whole-genome library RITseq screen that assessed loss of fitness in culture after 3 days of RNAi induction^12^ (Fig. S1A). Importantly for this study, reduced library size and associated RITseq resolution allowed a 72 h *in vivo* model of infection in mice, which would not have been possible with the larger parasite numbers required to cover the whole genome. Although whole genome RITseq screens may have lower resolution, they assess in parallel all the protein families, thus being a valuable tool to identify pathways and networks whose activity is required under any selective pressure or culturing condition.

Trypanosomes are highly adaptable organisms, capable of withstanding immune assault by the host and responding to the many changing conditions they experience during their life cycles, including temperature, pH, osmotic imbalance, shear-forces, and availability of carbon and nitrogen sources. This study reveals that depletion of at least nine PKs limits the capacity of *T. brucei* to replicate in the mouse bloodstream, while the same cells remain more competent for growth in culture, suggesting roles for the PKs as virulence factors required to overcome challenges encountered only *in vivo*. The precise functions provided by the PKs remain to be identified, but a common theme from sequence-based functional predictions is the management of stress. It has been shown in bacteria that, in addition to ‘direct’ or *sensu stricto* virulence factors (such as toxins or invasins), elements involved in stress management are also ‘indirect’ or ‘contributory’ elements required for establishment of the infection^19^. A connection between stress resistance and virulence has also been found for many other infectious eukaryotic organisms^20–24^, including *Leishmania* ^25, 26^. Sequence similarity between eight of the *T. brucei in vivo*-specific PKs and kinases in other eukaryotes suggests diverse functions, including gene expression, alternative splicing, protein synthesis and translational regulation in response to extracellular signals, and stress adaptation linked with autophagosome formation (Data set S4). One of the nine PKs differed from the rest, in that it appears to be a highly divergent, putative pseudo-kinase (Tb927.11.9290/FAZ20), since it lacks several of the key residues and subdomains required to be an active PK^27^. Previous work has shown that this PK localizes to the tip of the flagellum attachment zone in both the old and the new cell generated during cytokinesis^28^, perhaps suggesting parasite specific functions. Two MAP3K (STE11) PKs were identified amongst the *in vivo* cohort. Tb927.10.14300 (STE11/MRK1) has features in common with members of the yeast High Osmolarity Glycerol (HOG) pathway, which responds to osmotic challenge through actin recovery^29^, while the MORN motifs-containing protein Tb927.10.10350 (STE11/Bck1p) resembles human MEKK3, a positive regulator of the stress-activated protein kinase (SAPK) pathway^30^ putatively involved in cell integrity maintenance under stress conditions. The increased susceptibility to osmotic shock seen after RNAi induction of these two STE11 PKs (Fig. 4B) indicates that they may provide similar roles in *Trypanosoma brucei*. While human plasma is considered by convention isotonic, many tissues are exposed to osmotic fluctuations both physiological and triggered by inflammation for which all cells, including infectious pathogens, need to be prepared^31^. Immediate downstream interaction partners of STE11s (STE7s) were not identified in this screen, which may suggest redundancy amongst the *T. brucei* STE7 cohort, or perhaps a non-canonical mode of action for the *T. brucei* STE11 PKs. Two other MAPKs (ERK-like), often involved in transcriptional and non-transcriptional regulation in response to external stimuli, were found to be essential both *in vivo* and *in vitro*: KFR1, previously reported to be regulated by interferon γ in order to promote proliferation in *T. brucei* ^32^; and MAPK6 (ERK8), which regulates cytokinesis^33^.

Three PKs that have a pronounced loss of fitness *in vivo* cluster together within the PI3K/TOR pathway: Tb927.11.9190 (VPS15), Tb927.11.850 (Bud32) and Tb927.7.960 (SRPK1). VPS15 is required for stress-induced and developmentally-triggered autophagosome formation in other eukaryotes and assembles within the PI3K complexes^34^. PI3K signalling in yeast involves Bud32 (orthologues called PRPKs in humans and *Drosophila*), which acts as a transducer for TOR activation that, in turn, has also been related with autophagy and endocytosis under stress conditions. SRPK1 regulates splicing factors in response to stress and can be activated via PI3K signaling in other eukaryotes^35^. The identification of these TOR-related PKs is of interest, because TOR pathways are central for parasite-host interaction in *T. brucei* ^36^. While most eukaryotes encode two TOR paralogues, trypanosomatids are the only known organisms to contain four, each integrated in a different protein complex, of which three are required for normal fitness both *in vivo* and *in vitro* (this study and ref. 6). TbTOR1 regulates transcription and translation and its depletion leads to autophagy, thereby enhancing survival upon nutritional stress, which is linked to developmental progression^37^. TbTOR2 is required for actin polarization, which in turn is essential for secretion and endocytosis^37, 38^. TbTOR3 responds to osmotic shock by control of polyphosphates and acidocalcisomes^39^, organelles that are crucial for autophagy in *T. brucei* ^40^. The last paralogue, TbTOR4, is required for *T. brucei* proliferation and life stage maintenance in bloodstream forms as its depletion triggers differentiation from replicative slender to stumpy cell-cycle-arrested forms^41^. How the three *in vivo* only PKs identified here might act within the expanded PI3K/TOR signaling network awaits further analysis.

It is intriguing that three PKs with a pronounced loss of fitness *in vivo*, AUK2, CAMKL and SRPK1, have also been identified in a companion RNAi screen for factors required for *T. brucei* to withstand or repair damage caused by alkylation *in vitro* (Stortz *et al*., submitted). The three showed an increased sensitivity to serum upon RNAi induction, related to complement-mediated lysis. In what way, if at all, the two phenotypes might overlap is unclear but the potentially common function of each *T. brucei* PK in providing serum resistance (Fig. 4A) might guide future studies, and at least one (AUK2) clearly has a role in genome maintenance (Stortz *et al*., manuscript in preparation). The final gene that is only essential for *in vivo* growth, CK2A2, has validated protein kinase activity and localizes primarily to the nucleolus^42^. CK2 is mainly controlled by distribution and abundance, with stress-induced mobilization extensively reported^43^. CK2 has also been associated with autophagy through intersection with PI3K/AKT/TOR and MAPK pathways^44^.

*T. brucei* protein kinases are druggable^45^ and this work provides genetic validation for 49 protein kinases that are required for *in vivo* fitness. A phylogenetic comparative analysis between the *T. brucei* and *Homo sapiens* kinomes showed that 13 out of the 49 PKs had close human orthologues targeted by at least one of the compounds in the ‘*HAT box’* (Fig. ﻿3, data set S1 and ref. 8). Targets identified in this screen open an opportunity to develop drug repurposing strategies, as has been already suggested for aurora kinases^46^ and PI3K/TOR kinase inhibitors^47^. All of the 49 PKs important for *T. brucei* growth have close orthologues in either *T. cruzi*, *L. major* or both (Fig. S2). Extrapolation of results based on gene ontology using *T. brucei* as a model may be valuable to identify potential trypanosomatid-wide drug PK targets^48^, since to date these organisms lack efficient reverse genetics tools for kinome- or genome-wide studies.

## Materials and Methods

### Ethics statement

Animal procedures followed the guidelines and were approved by The Home Office of the UK government. The procedures here presented were covered by the project license PPL60/4442 entitled “Molecular Genetics of Trypanosomes and Leishmania”. The University of Glasgow ethics committee also approved all protocols.

### Parasite maintenance

Monomorphic *Trypanosoma brucei brucei* 2T1 bloodstream forms^49^ were cultured in HMI-11 [HMI-9 (GIBCO) + 10% v/v foetal bovine serum (GIBCO), Pen/Strep solution (penicillin 20 U ml^−1^, streptomycin 20 mg ml^−1^)] at 37 °C/5% CO_2_ in vented flasks. Selective antibiotics were used as follows: 0.2 μg ml^−1^ puromycin, 0.5 μg ml^−1^ phleomycin (InvivoGen), 2.5 μg ml^−1^ hygromycin B (Calbiochem). RNAi was induced *in vitro* with tetracycline (Sigma Aldrich) in 70% ethanol at 1 μg ml^−1^.

To test for serum resistance 200 μl of log-phase parasites in HMI11 at 10^6^ cells ml^−1^ were exposed to fresh rat serum for 3 h (10% and 50% concentration). Relative survival was measured of induced (for 48 h) and uninduced clones compared to no-serum controls. The whole experiment was performed in triplicate.

To test for response to osmotic shock^18^ 2 × 10^6^ cells were resuspended for 5 min at 4 °C in 0.5 ml of a solution 55 mM KCl/1 mM glucose to induce cell swelling. 0.5 ml of a 263 mM KCl/1.75 mM Mg_2_Cl shrinking solution was added on top and cells incubated for 5 min at 4 °C. Remaining cells were washed once and resuspended in 1 ml of HMI11 media before counting. Survival of the induced cells compared to uninduced controls was plotted and significant results (p < 0.05) after T-tests indicated with a star.

### RNAi library

All RNAi cell lines in this work were generated as previously described^6^. RNAi lines were pooled into 9 ‘*Mixed Stabilate Trypanosome Libraries*’ (MSTL 1–9), each containing 19–25 cell lines. Then MSTLs were further pooled to make the final culture.

The library used for the *in vitro* RITseq was defrosted, grown for 24 h and diluted to contain 1 × 10^5^ cells ml^−1^ in 100 ml. In triplicate the culture was split into two 50 ml flasks (tetracycline induced and uninduced control) and grown for 120 h, reducing cells to 1 × 10^5^ cells ml^−1^ every 24 h. 1 × 10^7^ cells were sampled daily for analysis.

### Animals and ***in vivo*** growth analysis

Female CD1 outbred mice (6–8 weeks old) were obtained from Charles River (Edinburgh, Scotland). Animals were given water and food *ad libitum*. Infections were performed by intraperitoneal injection and were evaluated daily by assessing parasitemia levels with a hemocytometer by tail vein sampling diluting blood in anticoagulant (CBSS/Heparin) 0.83% w/v. If parasitemia exceeded 10^8^ cells ml^−1^ mice were euthanized with carbon dioxide and cervical dislocation. RNAi was induced in mice with doxycycline hyclate (Sigma Aldrich) added in sugared drinking water (0.2 g/L plus 50 g/L sucrose). For the *in vivo* RITseq, blood from euthanized mice 72 h after infection was collected and immediately applied to DEAE-cellulose columns as described^50^. DNA was isolated from the parasites using a QIAamp DNA kit (Qiagen, Venlo, Netherlands).

### RNAi Target sequencing

The library pool used at Experiment 1 was the product of mixing MSTLs 1–8. A single universal cassette-specific primer (5′-TAATGCCAACTTTGTACAA**A**-3′) was used to PCR enrich RNAi inserts amplified from 10 ng of genomic DNA obtained per sample in a 50 µl reaction using Q5^®^ High-Fidelity DNA polymerase (NEB, Ipswich, USA). The PCR program was: 3 minutes at 98 °C; followed by 28 cycles comprising: 10 seconds at 98 °C, 10 seconds at 61 °C and 30 seconds at 72 °C; with a final extension step at 72 °C for 10 minutes. PCR products were cleaned up with Minielute PCR purification kit (Qiagen, Venlo, Netherlands), 400 ng of DNA per sample were fragmented, size selected (average read length after sequencing ~106 nucleotides) and processed according to Ion Torrent library prep protocols. The multiplexed sample was subjected to emulsion PCR and Ion Torrent sequencing. The library pool used at Experiment 2 was processed as above with the following exceptions, a mix of MSTLs 1–9, PCR annealing temperature was elevated to 64 °C, as additional 6 nucleotide-long labels were added to the single universal primers (list of oligonucleotides and multiplex distribution in Data set S5) enabling pooling of the samples and usage of a single commercial barcode.

### RITseq data analysis

Artificial chromosomes were generated *in silico* by concatenating the 183 amplicons representing all the RNAi targets included in this study, joining the 3′ end of one with the 5′ end of the next using a buffer sequence of 15 random bases. The coordinates of each sequence were recorded. The artificial chromosome sequences were indexed for use in Bowtie2 (short-read alignment software)^51^, using default parameters. For *in vivo* Exp.1, single end reads generated from each sample were selected by the presence of a 9 base diagnostic tag [GCCAACTTT] present within the universal primers, permitting 1 mismatch (insertion, deletion or substitution). Approximately 20% of the reads contained the RNAi cassette. For *in vivo* Exp.2 and *in vitro* RITseq, single end reads were identified and clustered according to 6 base-long barcodes before being processed as in Exp.1 (without the fragmentation/size selection step) finding 65–70% of reads containing the RNAi target. Selected reads were mapped to the artificial chromosomes with Bowtie2 (aligning in local mode). The “.sam” format files generated by Bowtie2 were parsed and the coordinates of the appropriate chromosome to which the read mapped were recorded. The mapped reads were assigned to the appropriate PK sequence by reference to the index generated above. The read was assigned if it lay entirely within the PK sequence or overlapped the 3′ or 5′ end of the PK sequence. In each replicate, accumulated read abundances were normalized by multiplying raw counts 10^6^ times, dividing by the sum of total valid reads accepted for the analysis in the whole sample and rounding to the next integer, i. e. reads per million (RPM). Bowtie2 mapping was used also to compare whole-genome/kinome-wide RITseq coverage as depicted in Fig. S2.

## Electronic supplementary material

Supplementary information

Dataset S1

Dataset S2

Dataset S3

Dataset S4

Dataset S5
