# Supplementary material for: RNAi screening identifies Trypanosoma brucei stress response protein kinases required for survival in the mouse
Source: Sci Rep. 2017 Jul 21;7:6156. doi: 10.1038/s41598-017-06501-8 (PMC5522463; doi:10.1038/s41598-017-06501-8)
Supplement: Supplementary file 1 — Supplementary information [file 41598_2017_6501_MOESM1_ESM.pdf]

RNAi screen identifies *Trypanosoma brucei* stress response protein kinases required for survival in the mouse

Fernando Fernandez-Cortes, Tiago D. Serafim, Jonathan Wilkes, Nathaniel G. Jones, Ryan Ritchie, Richard McCulloch and Jeremy C. Mottram.

Supplemental material

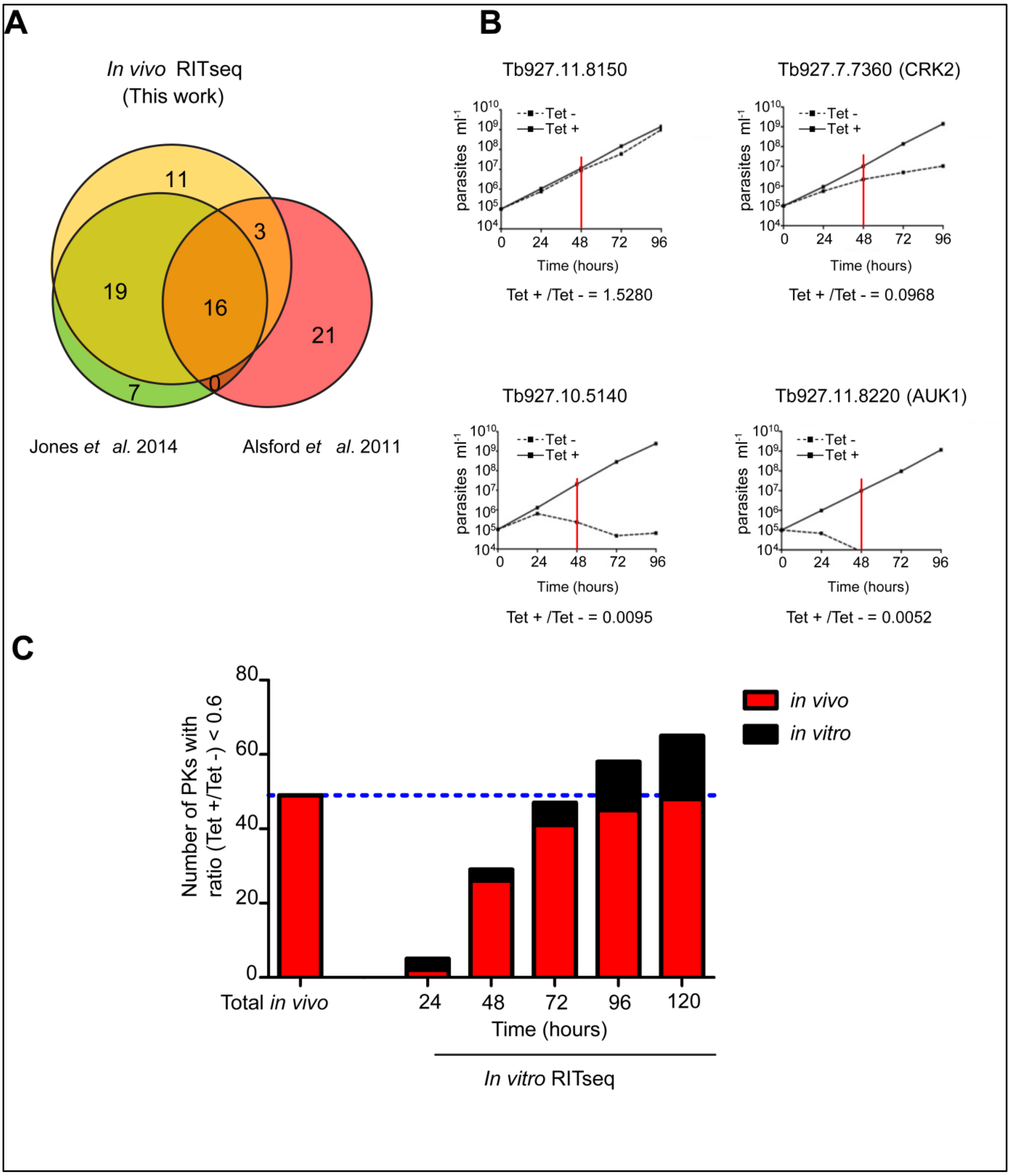

Figure S1. Comparison of *in vitro* and *in vivo* kinome-wide screens. A. Venn diagram showing the number of PKs with a loss of fitness in the present *in vivo* RITseq and two published *in vitro* RNAi studies after 72 h of induction[1,2]. **B.** *In vitro* growth curves for four independent RNAi cell lines with no growth defect (upper left), slow growth phenotype (upper right), growth arrest (lower left) and cell death (lower right). Median ratios of induced/uninduced

(Tet+/Tet-) for the *in vivo* RITseq (Table S1) are shown. **C.** Overlap between the number of cell lines with a loss-of-fitness phenotype detected *in vivo* after 48 h of induction and those detected with the *in vitro* RITseq over 120 h of RNAi.

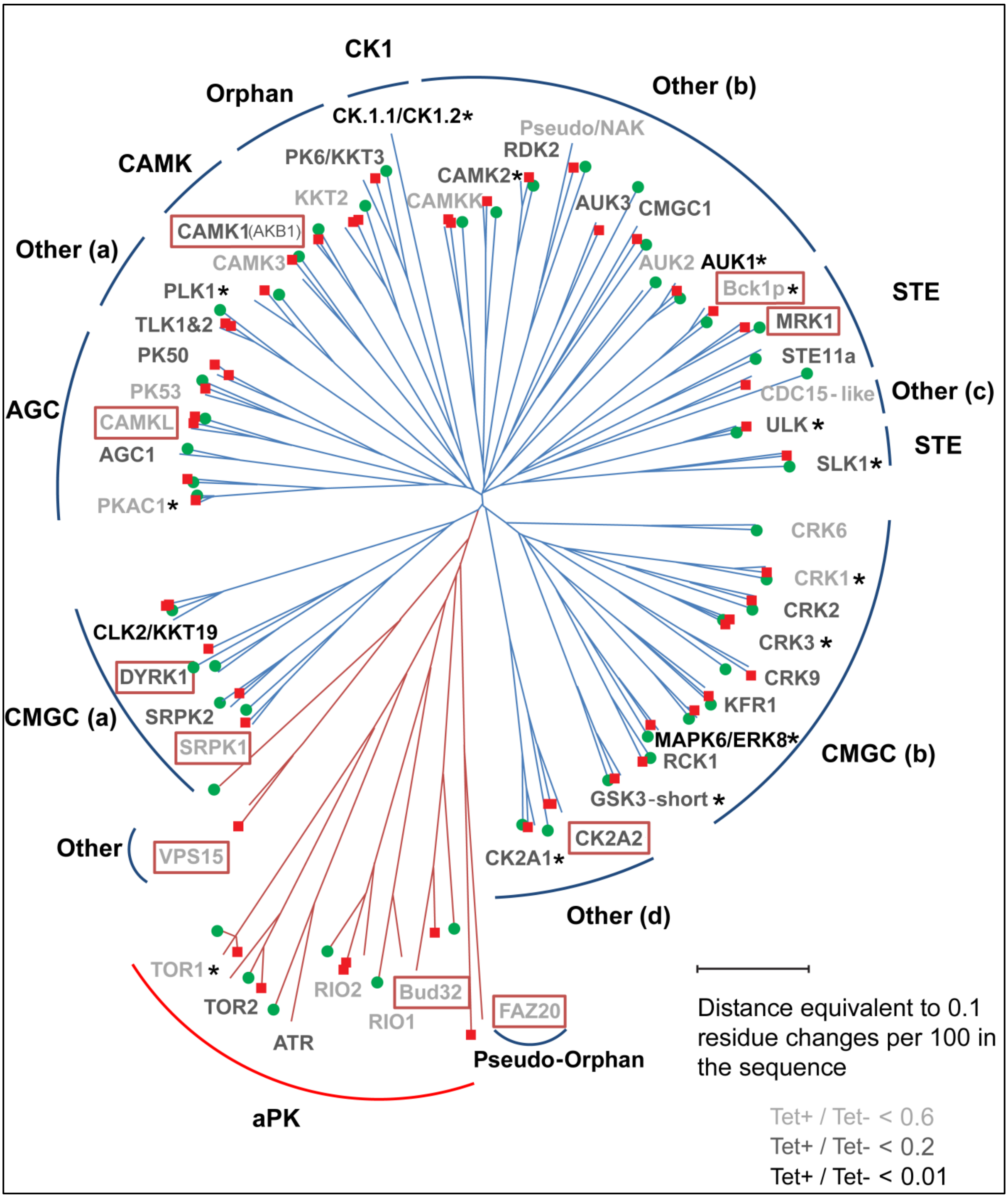

Figure S2. Neighbour-Joining phylogenetic tree of *T. brucei* protein kinases showing RNAi dependent loss-of-function *in vivo* with their *T. cruzi* and *L. major* orthologues. Red bars represent aPKs and blue bars ePKs. PK lines causing a phenotype *in vivo* not reported before *in vitro* [1,2] after RNAi induction are inscribed within a red box; those with a human orthologue

targeted by a compound in the HAT box [3] are labelled with a “*”; *L. major* and *T. cruzi* orthologues are labelled with a green circle and a red square respectively. Grey scale described intensity of the loss of fitness according to the mean Tet+/Tet- ratio.

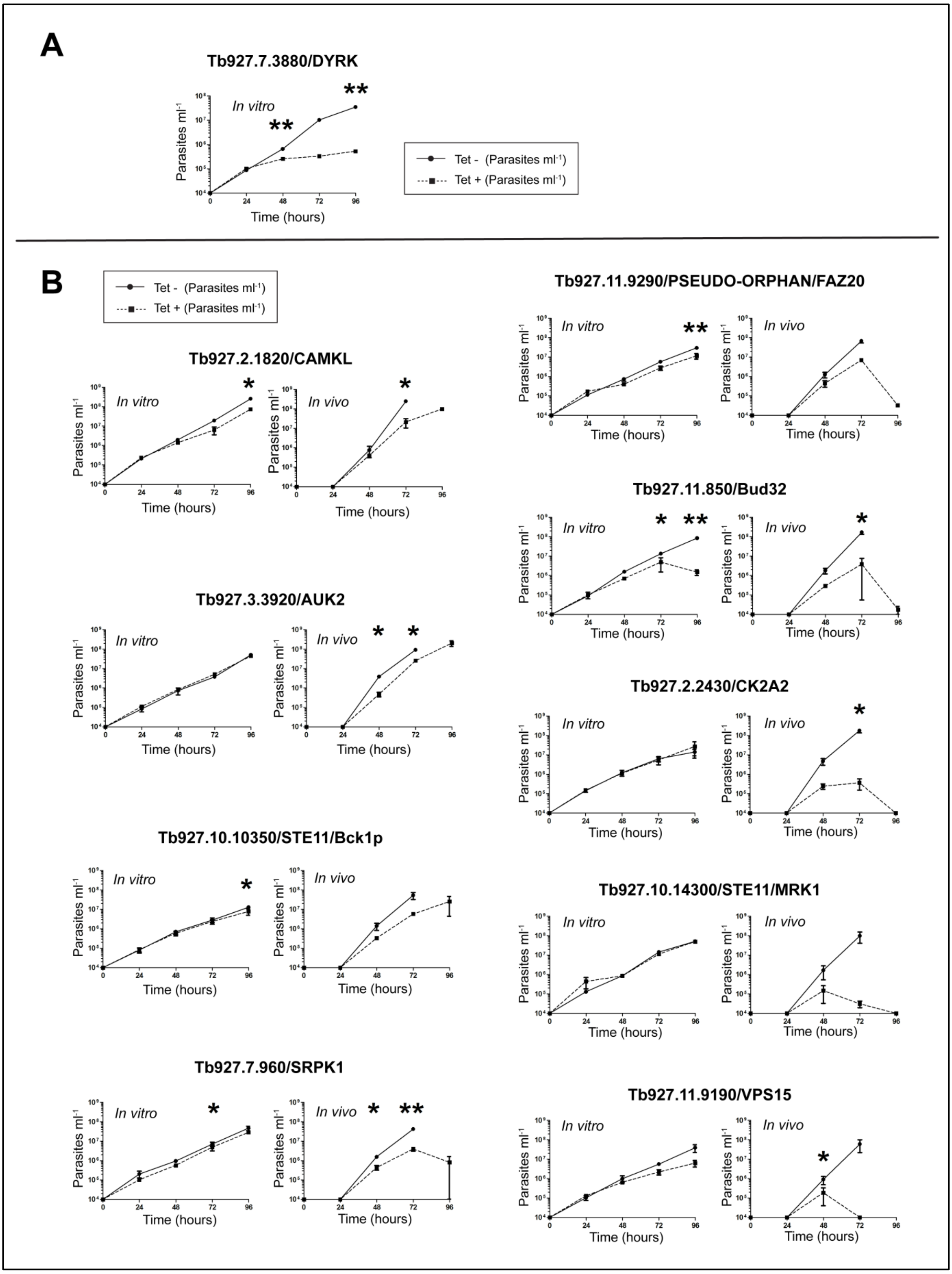

Figure S3. Validation of RITseq *in vivo* loss of fitness phenotypes. For RNAi cell lines, cumulative *in vitro* growth curves are shown on the left hand side; and *in vivo* parasitemia on the right hand side. Growth curves were built in triplicate.

In vitro cultures started at 10^4 cells ml^{-1}. Mice were culled if parasitemia reached 10^8 cells ml^{-1}. **A.** Tb927.7.3880 (DYRK). **B.** 9 other cell lines with a loss of fitness phenotype *in vivo* that is more pronounced than the one observed *in vitro*. * $p < 0.05$ and ** $p < 0.01$ using T-test.

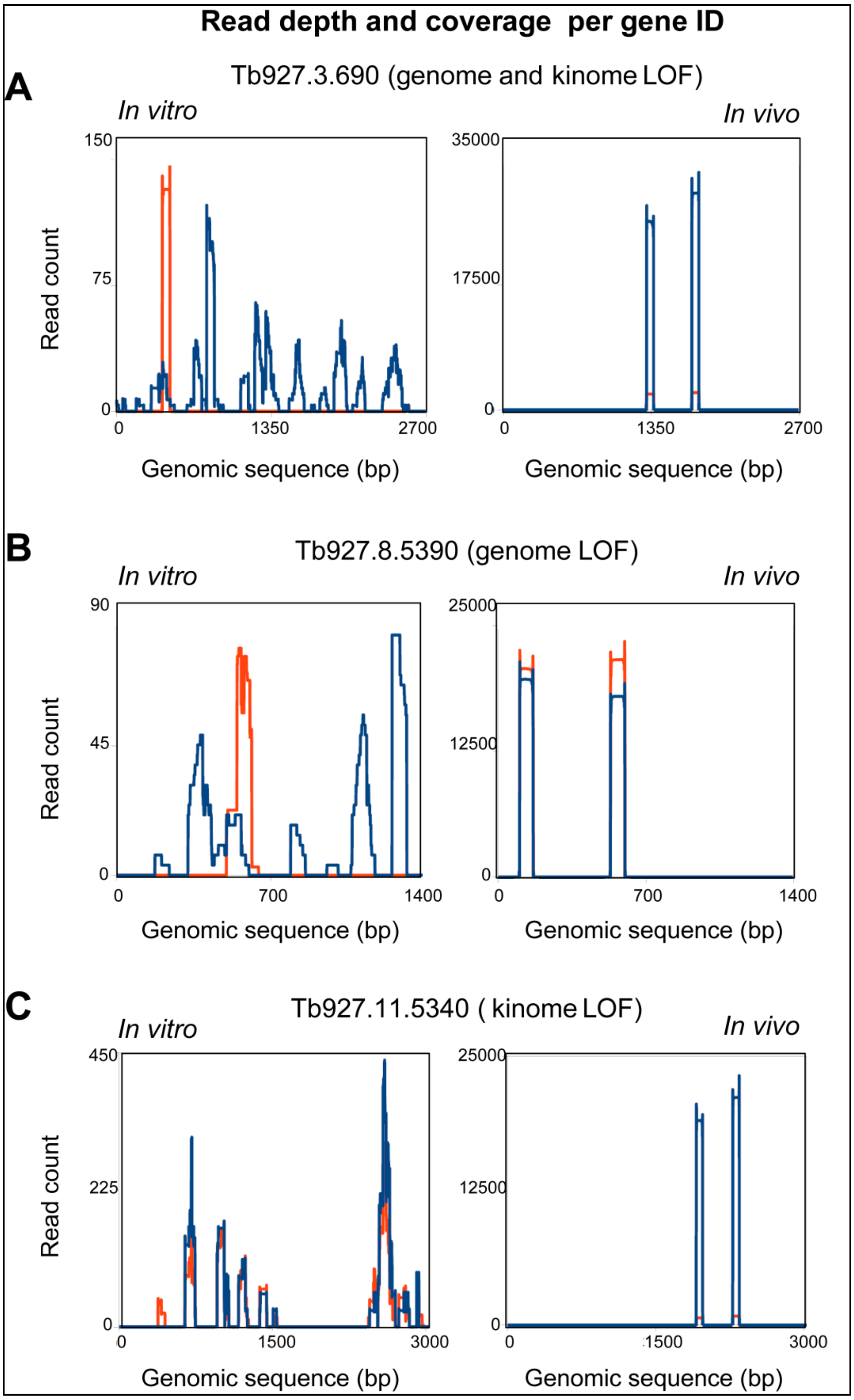

Figure S4. Comparative kinome focused/whole genome RITseqs [2]. A. Compared read count resolution for a gene that causes loss of fitness (LOF) both in the kinome-wide and in the genome-wide screening (Tb927.3.690); **B.** for a gene

that is a LOF only in the genome-wide (Tb927.8.5390); and **C.** for a gene that is a LOF in the kinome-wide (Tb927.11.5340).

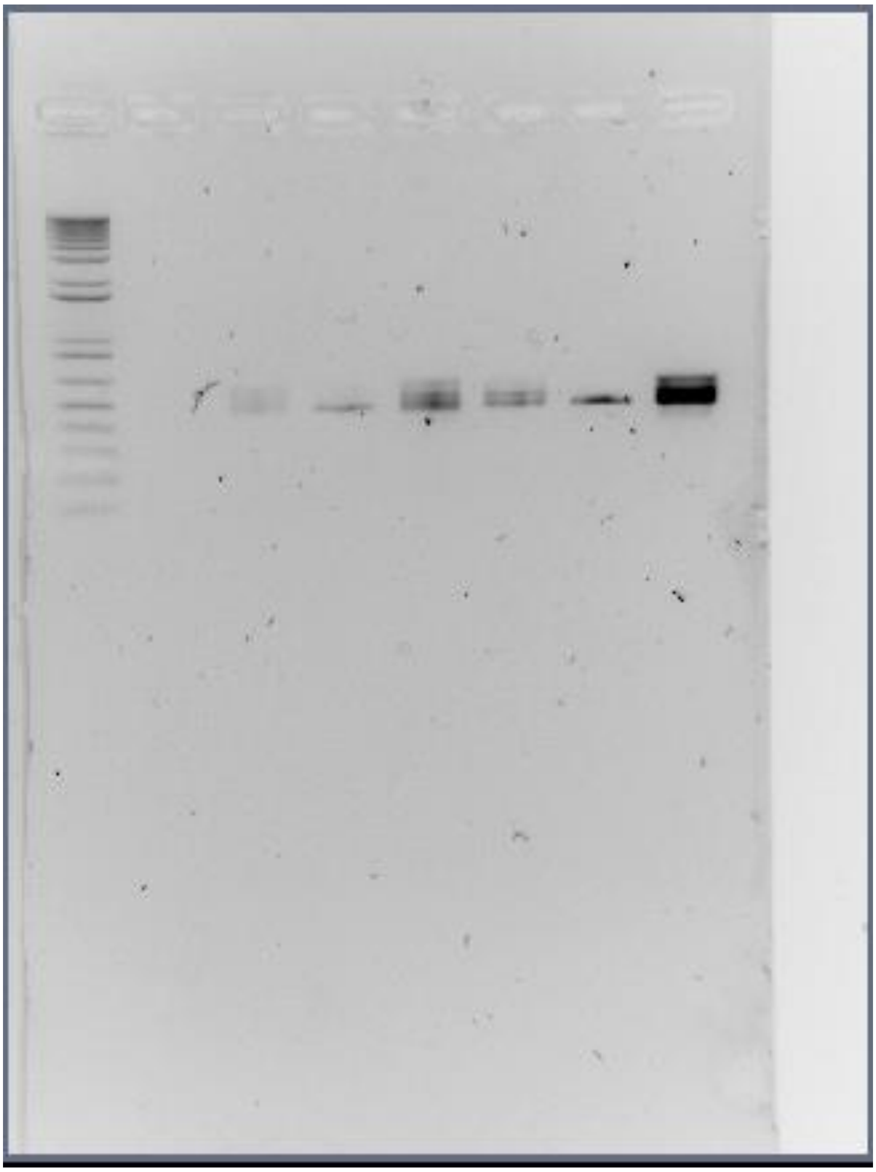

Figure S5. Complete uncropped gel depicted in figure 1.

Data Set S1. RITseq summary of results *in vivo* and *in vitro*. *Tab 1.* Listing of all detectable *T. brucei* lines noting PK family and name. Including: sub-library used to pool the cell line (column D); bootstrap median ratios (induced/control) for each of the two *in vivo* biological replicates and average between them (columns E-G); bootstrap median ratios (induced/control) for each of the *in vitro* time points (columns H-L); phenotype observed at the *in vitro* 72h kinome-wide screen by Jones et al [1]: growth ability (column M) and

cell cycle phenotype (column N); loss of fitness observed at the *in vitro* genome-wide screen by Alsford et al [2] noting induced/control ratio (left) and significance (right) after 3 days (columns O and P), and 6 days (columns Q and R) of RNAi induction. ***Tab 2*****. Meta-analysis for the 49 genes that had a phenotype *in vivo***. Genes reported in the literature (column D); sub-library used to pool the cell line (column E); evolutionary relation to identified human targets for compounds available in the HATbox [3] (column T); and orthologues in *L. major* (column U) and *T. cruzi* (column V).

Data set S2. Complete RITseq *in vitro* dataset. *Tab 1*. Summary of bootstrap median ratios control/input (columns B-F) and induced/control (columns G-K). ***Tabs 2-6***. One tab per time point. Raw read counts for input, control and induced replicates (columns B-J); normalized counts: multiplied 10^6 times and divided by sum of total reads rounded to the next integer (columns L-T); median control/input ratios after 1000 bootstrap replicates including limits of the 95% interval of confidence (columns V-X); and median induced/control ratios after 1000 bootstrap replicates including limits of the 95% interval of confidence (columns Z-AB).

Data set S3. Complete RITseq *in vivo* dataset. *Tabs 1-2*. One tab per biological replicate (Ion Torrent and Illumina Nextseq). Raw read counts for input, control and induced replicates (columns B-P); normalized counts: multiplied 10^6 times and divided by sum of total reads rounded to the next integer (columns R-AE); and median induced/control ratios after 1000 bootstrap replicates including limits of the 95% interval of confidence (columns AG-AI).

Data set S4. Putative function based in gene ontology for the 9 PKs causing a loss of fitness only *in vivo*. BLASTp analysis against *T. brucei, T. cruzi*, *Leishmania major*, *Saccharomyces cerevisiae*, *Caenorhabditis elegans, Drosophila melanogaster* and *Homo sapiens.* Displayed percentage of sequence which is identical, E score and putative function after literature search. References used annotated under the table.

Data set S5. Barcoded primers for illumina sequencing runs. Fourteen barcoded oligonucleotides used for PCR enrichment at the *in vitro* RITseq and the second *in vivo* RITseq, Highlighting samples where they were used, and Illumina multiplexed samples where they were pooled.

Supplementary materials and methods

Parasite maintenance

In vitro growth curves were performed with parasites at initial concentration of 1 x 10^4 cells ml^{-1} and evaluated daily over four days. After 48 h parasites reached ~10^6 cells ml^{-1}, so in order to maintain the log phase of growth they were diluted 1:100. For cumulative growth, a dilution factor of 100 was applied. Significant changes induced/control were assessed performing T-tests.

RNAi library

This intermediate step reduced risks of overgrowth and cross contamination. To make the MSTL pools, independent cell lines were defrosted diluting glycerol to 1%

with HMI-11. After 24 h recovery at 37 ^{o}C and 5% CO_2, cells were diluted again adding selective drugs (hygromycin and phleomycin). After 24 h, cultures were set at 2 x 10^5 cells ml^{-1} and after a further 24 h they were counted and pooled at 6 x 10^6 cells per PK in a common flask that was diluted to 50 ml. Cells were allowed to acclimatize for 1h at 37°C and the mixed culture was centrifuged 1500xg for 10 minutes. Parasites were resuspended in 24 ml of HMI-11, 10% glycerol to generate 24 cryostabilates per MSTL.

The final pool was made by mixing equivalent cell numbers from each MSTL sub-library. The culture was acclimatized at 37 ^{o}C 5% CO_2 for 45 min, cells pelleted at 1200xg for 10 min and resuspended at 5 x 10^5 cells ml^{-1}. After 6 h incubation cells were counted, washed and resuspended with PBSG to produce 200 µl inoculums of 5 x 10^4 cells ml^{-1}.

Phylogenetic tree

Coding sequences of 49 PKs with a loss-of-fitness phenotype and their *Trypanosoma cruzi* and *Leishmania major* orthologues were extracted from the TritrypDB platform [4]. They were all aligned against the PK hidden Markov model (HMM), PF00069 (obtained from PFAM [5], using the Hmmer application hmmalign [HMMER 3.1 (February 2013); http://hmmer.org/]) and trimmed to include only sequences conformant with the profile. The resulting clustalw file was converted to ungapped FASTA format. These conformant sequences were aligned using three additional and complementary programs: T-coffee [6], ClustalW [7] and MUSCLE [8]. Results obtained from all three alignment tools were combined in T-coffee and the output was processed to remove low quality (<5) and low occupancy (<60%)

regions. The resulting high quality alignment was used in Splits Tree [9] to generate the final Neighbour-Joining phylogenetic tree.

List of references

1. Jones, N. G. *et al.* Regulators of *Trypanosoma brucei* cell cycle progression and differentiation identified using a kinome-wide RNAi screen. *PLoS Pathog.* **10,** e1003886 (2014).

2. Alsford, S. *et al.* High-throughput phenotyping using parallel sequencing of RNA interference targets in the African trypanosome. *Genome Res.* **21,** 915–924 (2011).

3. Peña, I. *et al.* New Compound Sets Identified from High Throughput Phenotypic Screening Against Three Kinetoplastid Parasites: An Open Resource. *Sci. Rep.* **5,** 8771 (2015).

4. Aslett, M. *et al.* TriTrypDB: a functional genomic resource for the Trypanosomatidae. *Nucleic Acids Res.* **38,** D457–D462 (2010).

5. Finn, R. D. *et al.* Pfam: the protein families database. *Nucleic Acids Res.* **42,** D222–D230 (2014).

6. Notredame, C., Higgins, D. G. & Heringa, J. T-coffee: a novel method for fast and accurate multiple sequence alignment. *J. Mol. Biol.* **302,** 205–217 (2000).

7. Larkin, M. a. *et al.* Clustal W and Clustal X version 2.0. *Bioinformatics* **23,** 2947–2948 (2007).

8. Edgar, R. C. MUSCLE: multiple sequence alignment with high accuracy and high throughput. *Nucleic Acid Res.* **32,** 1792–1797 (2004).

9. Huson, D. H. & Bryant, D. Application of phylogenetic networks in evolutionary studies. *Mol. Biol. Evol.* **23,** 254–267 (2006).
